# Inhomogeneity of the Backward Extruded NdFeB Ring Magnet Prepared from Amorphous Powders

**DOI:** 10.3390/ma16145117

**Published:** 2023-07-20

**Authors:** Weida Huang, Ke Xu, Xuefeng Liao, Bang Zhou, Hongya Yu, Xichun Zhong, Zhongwu Liu

**Affiliations:** 1 School of Materials Science and Engineering, South China University of Technology, Guangzhou 510640, China; huangweida2000@163.com (W.H.); 18813298924@163.com (K.X.); 18846321367@163.com (B.Z.); yuhongya@scut.edu.cn (H.Y.); xczhong@scut.edu.cn (X.Z.); 2 Institute of Resources Utilization and Rare Earth Development, Guangdong Academy of Sciences, Guangzhou 510650, China; liaoxuefeng@126.com

**Keywords:** radially oriented NdFeB ring magnets, backward extrusion, amorphous powders, magnetic properties, microstructure

## Abstract

Back extrusion is an important process to prepare radially oriented NdFeB ring magnets. In this work, we fabricate the ring magnets using amorphous magnetic powders as the raw material. The microstructure, magnetic properties, corrosion resistance, and mechanical properties of the backward extruded magnet at different positions along the axial direction have been investigated, and the inhomogeneity of the magnet is clarified. The results showed that the grains in the middle region of the ring magnet exhibit a strong *c*-axis orientation, whereas the grains at the bottom and top regions are disordered with random orientation. The microstructure variation is related to the distribution of the grain boundary phase and the degree of grain deformation. Due to the microstructure difference, the magnetic properties, temperature stability, corrosion resistance, and mechanical properties in the middle region of the magnet are higher than those in the top and bottom regions. The exchange coupling between grains also varies in different regions, which is related to the grain size and grain boundary thickness. In addition, different Co element segregations were observed in different regions, which has a crucial effect on the Curie temperature and thermal stability of the magnet. The microstructure difference also leads to the variation of corrosion resistance and mechanical properties for the samples from different regions of the magnet. This work suggests that the amorphous powder can be used to directly prepare radially oriented ring magnets, and the inhomogeneity of the magnet should be fully understood.

## 1. Introduction

Radially oriented NdFeB ring magnets are ideal permanent magnets used in various electric motors, such as servo motors, EPS motors, and drive motors, due to their high magnetic flux and radial orientation [1]. Currently, there are three different approaches to preparing radially oriented ring magnets, including sintering, bonding, and hot deformation [2]. However, each method has its own drawbacks. The sintering process requires a radial magnetic field to align the grains, and the shrinkage during preparation can lead to inaccurate shape control [3]. The bonding process results in well–formed magnets, but their magnetic properties are low due to their isotropic structure [4]. In contrast, the backward hot extrusion method, as a special hot deformation process, can be directly used to prepare radially oriented NdFeB ring magnets with good magnetic properties. Due to its simple process and good formability, this approach has been initially industrialized [5,6].

During the backward extrusion process of NdFeB magnets, preferential orientation of the main phase Nd_2_Fe_14_B grains occurs under pressure, accompanied by grain boundary sliding and rotation, resulting in *c*-axis alignment in the radial direction and the gradual growth of platelet–like grains [7]. The molten low melting point grain boundary phase can act as the lubricant, aiding the upward creep of the main grains along the gap between the punch and the mold, ultimately resulting in the formation of the radially oriented ring magnet. The backward extrusion method has been found to be able to produce ring magnets with thin wall thickness, small diameter, high aspect ratio, and excellent magnetic properties [8]. Until now, numerous backward extruded NdFeB ring magnets produced from nanocrystalline magnetic powders have been reported with various magnetic properties. Gutfleisch et al. [9] successfully prepared the ring magnets with properties of *J_r_* = 1.07 T, *H_cj_* = 575 kA/m and *(BH)_max_* = 206 kJ/m^3^ using HDDR magnetic powder with the composition of Nd_15_Fe_77_B_8_. Li et al. [10,11] used Nd_30.5_Fe_bal_Co_6.0_Ga_0.6_Al_0.2_B_0.9_ (wt.%) melt-spun powder as a starting material and improved the magnetic ring performance to *J_r_* = 1.27 T, *H_cj_* = 725 kA/m, and *(BH)_max_* = 302 kJ/m^3^ by optimizing the preparation process and magnetic powder composition. Despite this progress, this process is also facing some practical problems, such as difficulties in demolding and large–scale production. Most importantly, the extruded magnets generally exhibit inhomogeneous microstructure and properties, which is not friendly to industry production [12,13,14,15,16]. However, systematic research on the inhomogeneity of backward extruded NdFeB ring magnets is still lacking.

We recently reported a process study on the preparation of hot–pressed (HP) and hot-deformed (HD) NdFeB magnets by amorphous and nanocrystalline powders [17], and it was found that higher hard magnetic properties can be obtained in the magnets prepared by amorphous powders. Directly using melt–spun amorphous powder as the precursor for HD magnets is an energy-saving process since no heat treatment is needed for the powders. However, up to now, no systematic research has been conducted on the backward extruded NdFeB magnets prepared from amorphous powders.

Thus, in this work, we investigated the preparation of radially oriented NdFeB ring magnets using backward extrusion with amorphous magnetic powder. The microstructure and properties at the different parts of the radially oriented ring magnets were analyzed in detail. The reasons for the inhomogeneity in the magnet were clarified.

## 2. Materials and Methods

The commercial amorphous Nd–Fe–B powders with the composition of Nd_28.47_Pr_0.56_Fe_bal_B_0.97_Co_3.63_Al_0.13_Nb_0.56_Zr_0.44_ (wt.%) were used as raw materials, and the powder particle size is 45–250 µm. The fully dense cylindrical precursor with a size of Φ20 × 10 mm was prepared by hot-pressing in a vacuum at 700 °C and 400 MPa for 30 min. The precursor was sanded and placed in a homemade backward extrusion mold and subsequently extruded at 750 °C with an average deformation rate of 2 × 10^−2^ s^−1^ to obtain radially oriented ring magnets. The dimension of the ring magnet is 20 mm outer in diameter, 15 mm in inner diameter, 2.5 mm in wall thickness, and 15 mm in height.

The magnetic properties of the Φ2 × 2 mm samples cut from the ring in different regions along the axial direction were measured by a vibrating sample magnetometer (VSM, PPMS–9, Quantum Design, San Diego, CA, USA) with a maximum magnetic field of 5 T. The density of each sample was measured using a high–precision metal density tester (AR-150ME, Shanghai Jingqi Instrument Co., Ltd., Shanghai, China). The demagnetization correction was carried out based on the sample geometries. Grain alignment was characterized by X–ray diffraction (X’ Pert Pro, PANalytic, Almelo, The Netherlands) with Cu K_α_ radiation. The microstructures were investigated by a scanning electron microscope (Quanta200, FEI, Hillsboro, OR, USA) and a transmission electron microscope (TEM, JEM-1400plus, JEOL, Tokyo, Japan) equipped with an energy dispersive spectrometer (EDS, X-MaxN 80T, Oxford, England). The polarization curve of the samples was measured by an electrochemical workstation (Autolab-PGSTAT302N, Metrohm, Herisau, Switzerland) in 3.5 wt.% NaCl solution in a scan rate of 2 mV/s. The mechanical properties were measured by universal mechanical testing machine (AG-100NX, Shimadzu, Shanghai, China) with a loading load speed of 0.3 mm/min. The loading direction was parallel to the *c*-axis direction of the sample.

## 3. Results

### 3.1. Phase Analysis and Grain Orientation

**Figure 1:**
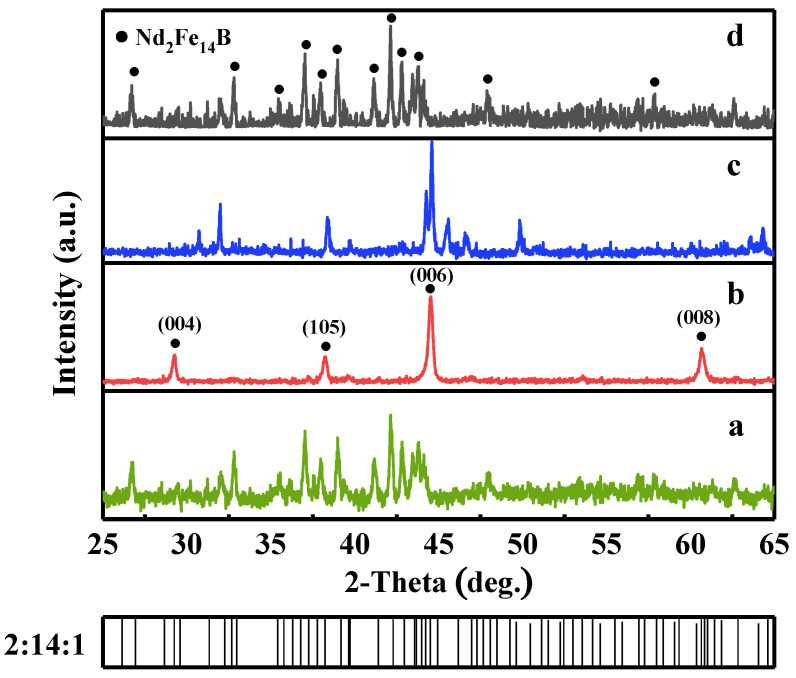
XRD patterns of the extruded ring magnet, where pattern a, b, c, and d were obtained from the bottom to the top in different regions of the magnets, detected from the plane perpendicular to the radial direction.

The XRD patterns for the different regions of the backward extruded magnet from the bottom to the top in the order of a, b, c, and d are shown in Figure 1. The detecting plane is perpendicular to the radial direction. The results show that the main phase of the magnet is the Nd_2_Fe_14_B, i.e., 2:14:1 phase, and no other phases are detected. In the bottom and top regions of the ring, the crystalline diffraction peaks with large angles to (00*l*) appear, such as (214), (115), (411), and (333), are detected, indicating the grains are not well aligned. In the middle region of the ring, the diffraction intensity of the characteristic peaks such as (006) and (105) is significantly enhanced, and other spurious peaks are decreased, indicating the *c*–axis along the (00*l*) direction. To compare the texture degree of (00*l*), the intensity ratio of (006) and (105) peak, denoted as *I*_(006)_/*I*_(105)_, is usually used [18]. The *I*_(006)_/*I*_(105)_ peak intensity ratios are calculated to be 3.64 and 3.32 for the samples in the central b and c regions, respectively, compared to 1.11 and 1.07 for those in the a and d regions. Hence, the result clearly shows that in the middle regions of the ring, the magnet exhibits higher radial grain *c*-axis orientation and strong magnetic anisotropy.

As we know, the grain boundary RE–rich phase in NdFeB magnets has an important effect on grain orientation [19]. At elevated temperatures above 650 °C, the grain boundary RE-rich phase melts into liquid, which can wet the grain boundary and wraps the main grains. During the backward extrusion, the grains can rotate freely, and grow selectively under radial pressure, further forming *c*–axis oriented platelet–like grains. However, in the top region of the ring, the extrusion pressure is too small and the extrusion time is not enough, resulting in insufficient deformation of the main grains. Most of the grains at the bottom of the ring magnet fail to reach the extrusion deformation zone and therefore are not effectively oriented. Hence, these two regions exhibit a non–uniform distribution of grain boundary phase and irregular grain shape, leading to grains that are not well-oriented.

### 3.2. Microstructure

**Figure 2:**
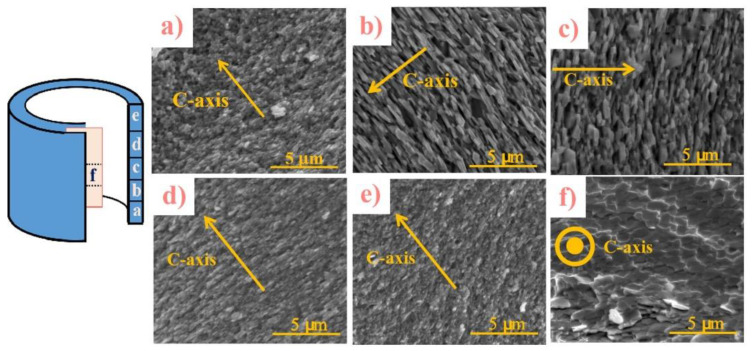
SEM images of the extruded ring magnet from the bottom to the top in different regions (**a**–**e**) parallel to the pressing direction and the cross–section of the middle region (**f**).

Figure 2 shows the SEM images of the backward extruded magnet in different regions parallel to the pressing direction. The morphology from the bottom to the top regions a, b, c, d, and e are shown in Figure 2a, b, c, d and e, respectively. It is clear that in the middle of the ring, the grains are arranged in a stacked platelet–like structure, and the aspect ratio of the grain is large, as shown in Figure 2b–d. The cross-section image of the middle region is shown in Figure 2f, which confirms that the grains are lamellar in distribution with the *c*–axis perpendicular to the direction of pressure and pointing out of the plane. However, in the bottom and top of the magnet, the aspect ratio of the grains is relatively small, and the grains are non–oriented and disordered equiaxed, as shown in Figure 2a,e. Combined with the XRD results, the microstructure of the ring from top to bottom is inhomogeneous. In the middle region of the ring, the grains are platelet–like with a well–radial orientation along the c-axis direction.

**Figure 3:**
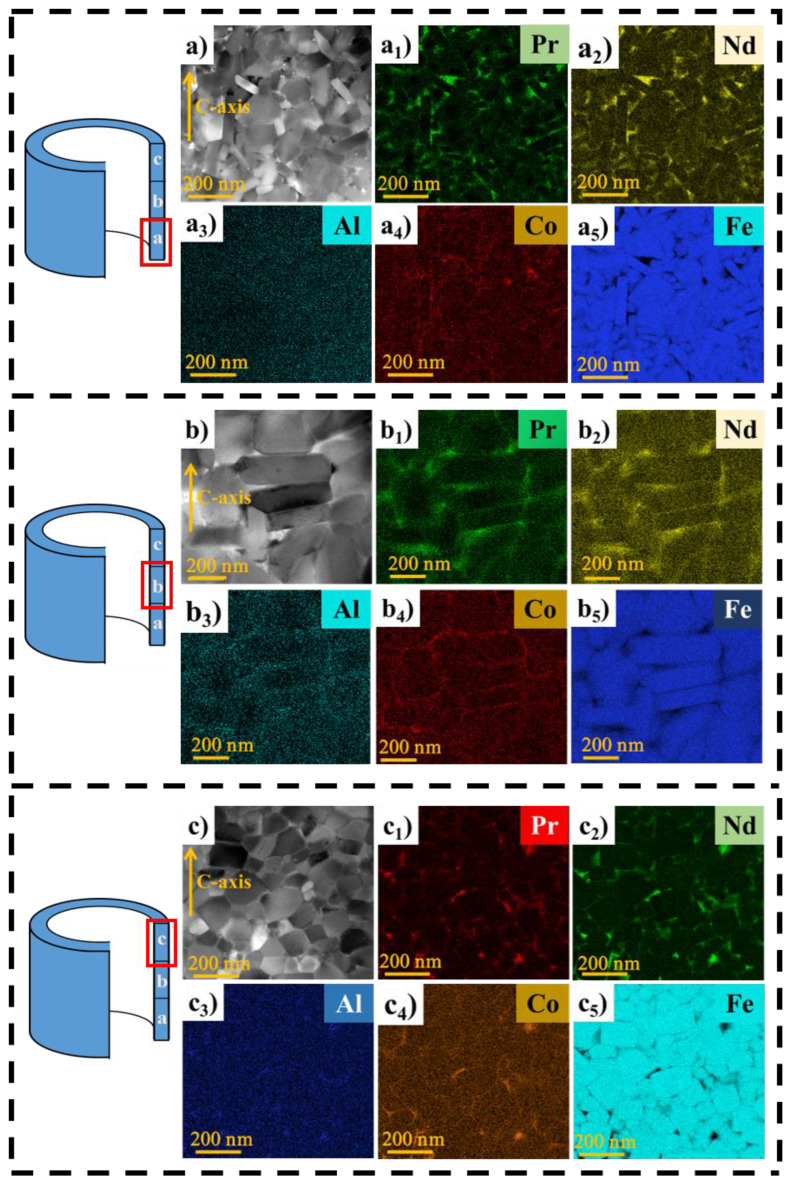
TEM images and EDS mapping of the bottom (**a**–**a_5_**), middle (**b**–**b_5_**), and top (**c**–**c_5_**) regions of the extruded ring magnet parallel to the pressure direction surface.

Figure 3 gives the TEM images for the microstructure of extruded ring magnets in different regions and the corresponding EDS elemental mapping results. Based on the TEM images, the average grain sizes in different regions of the ring parallel to the *c*-plane (D_//_) and perpendicular to the *c*-plane (D_⊥_) were measured, and the results are shown in Table 1. As shown in Figure 3a–a_5_, the disordered and weak-oriented equiaxial grains are evident in the bottom region. The average sizes D_//_ and D_⊥_are ~123 nm and ~74 nm, respectively. The Fe element is mainly enriched in the main phase grains. Pr and Nd elements are enriched in the grain boundaries, forming RE–rich phases, which is beneficial to magnetically isolate the main grains. Figure 3b–b_5_ show that in the middle region of the ring, the grains are lamellar platelet-like in shape with an average size of ~336 nm in length and ~120 nm in width. The aspect ratio of the grain has increased compared to that in the bottom region. The grain boundary phase is also Pr– and Nd–rich, but the thickness of the grain boundary is relatively thin, which will weaken the isolation of the main grains and enhance the role of exchange coupling, resulting in a lower coercivity of the magnets. Figure 3c–c_5_ show that in the top region of the magnet, the grains are disordered and unoriented with an equiaxial shape. The average grain sizes D_//_ and D_⊥_ are ~94 nm and ~78 nm, respectively. The finer grains in the top region are believed to be beneficial for enhancing coercivity, according to the empirical relationship between µ_0_H_c_ and average grain size reported by Kronmüller et al. [20].

In addition, Figure 3 also shows that in the bottom region (Figure 3a_4_) and top region (Figure 3c_4_), the Co element is enriched in grain boundaries. However, in the middle region of the magnet, the Co element partially enters into the main phase rather than being completely distributed in the grain boundary phase. The result suggests that the segregation of the Co element is influenced by the grain morphology, which is actually determined by the deformation degree.

### 3.3. Magnetic Properties

**Figure 4:**
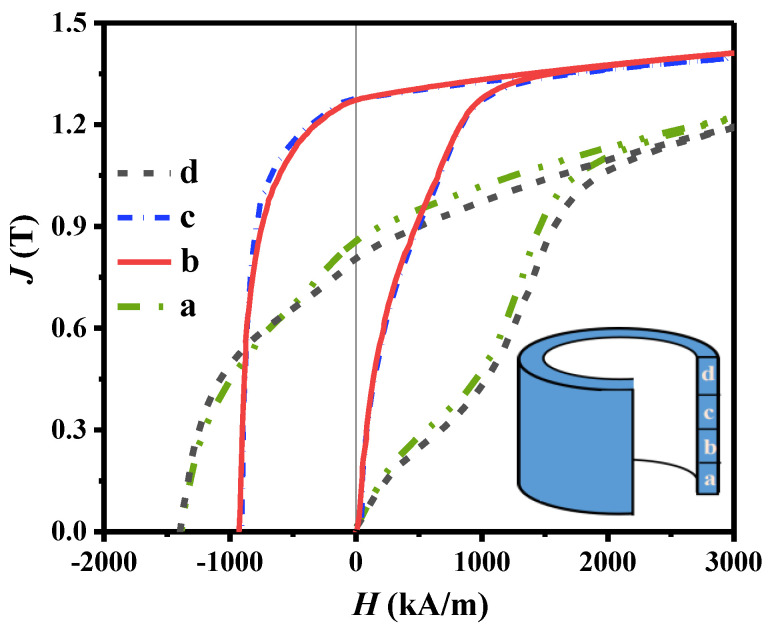
Demagnetization curves of the extruded ring magnet, where line a, b, c, and d were obtained along the radial direction from the bottom to the top in different regions.

The room−temperature initial magnetization and demagnetization curves of backward extruded magnets in the radial direction from the bottom to the top in different regions are shown in Figure 4. The density and magnetic properties for different regions are shown in Table 2. The densities of the different regions of the ring range from 6.9 g/cm^3^ to 7.5 g/cm^3^. Since the extrusion pressure varied in different regions during the deformation of the ring, the density is not uniform in the magnet.

The bottom of the magnet (region a) has magnetic properties with *J_r_* of 0.86 T, *H_cj_* of 1386 kA/m, and *(BH)_max_* of 111 kJ/m^3^. The top of the ring (region d) shows *J_r_* of 0.85 T, *H_cj_* of 1410 kA/m, and *(BH)_max_* of 127 kJ/m^3^. The magnetic properties of these two regions are close to hot-pressing magnets prepared by amorphous magnetic powders in previous studies [17], indicating that the bottom and the top grains of the ring are not sufficiently deformed. In the middle of the magnet, the magnetic properties of region b and region c are *J_r_* of 1.27 T, *H_cj_* of 930 kA/m, *(BH)_max_* of 270 kJ/m^3^, and *J_r_* of 1.28 T, *H_cj_* of 920 kA/m, *(BH)_max_* of 270 kJ/m^3^, respectively. The magnetic properties in the central regions of the ring magnet do not differ significantly, indicating that its microstructure is uniform in this region. The M_r_/M_s_ values of these two regions are greater than 0.912, which indicates that the magnetization axis is the same as the magnetization direction, and the grains are well−oriented along the *c*−axis. However, the coercivity in the middle region is relatively low. The reason should be attributed to the grain orientation. The middle region of the magnetic ring is subjected to compressive stress from the radial direction caused by the extrusion from the top, and the grains can be sufficiently aligned in the radial direction. Wang et al. [21] simulated the extrusion process of the magnetic ring and found that the grains in the middle region had a higher effective strain. Hence, the grains in the middle region have a higher *c*−axis orientation. The top region of the magnetic ring is subjected to insufficient extrusion pressure, and the grains are not sufficiently deformed to obtain orientation. At the bottom of the ring, there are multi−directional extrusion stresses in the region near the punch, and a part of the grains are randomly oriented along these directions. The *c*−axis of these grains would deviate from the radial direction, resulting in a decreased orientation. Thus, compared with the bottom and top regions of the magnet, the remanence increases significantly, and the coercivity decreases slightly.

In addition, Figure 4 also shows that the shape of the initial magnetization curve for the magnet in different positions is quite different. The initial magnetization curves of the bottom and top regions of the ring are *S*−shaped, and the coercivity mechanism in these two regions is a pinning type. In the middle part, the initial magnetization curve shows a large susceptibility, and the pinning effect gradually decreases.

**Figure 5:**
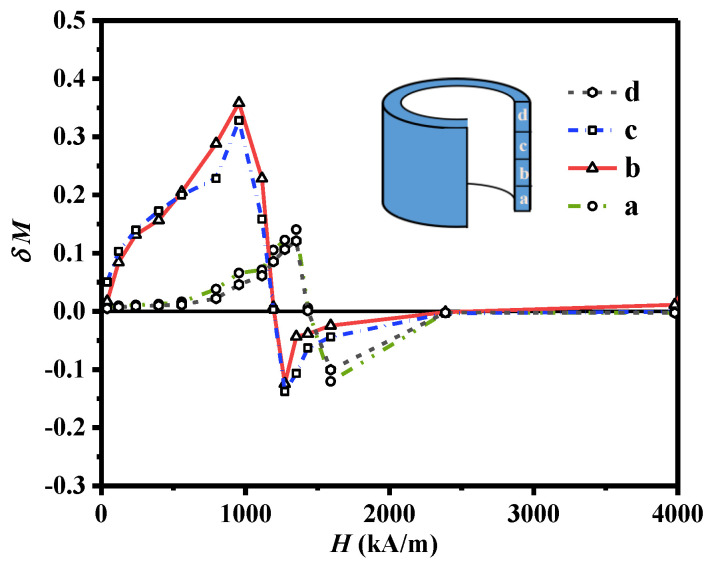
The Henkel curves of the extruded ring magnet, where line a, b, c, and d were obtained along the radial direction from the bottom to the top in different regions.

The above results show that the magnetic properties of the ring magnet are inhomogeneous along the axial direction, and the middle region of the ring has better magnetic properties. The magnetic properties of magnets are related to the microstructure and inter−grain exchange coupling. The Henkel curves proposed by Kelly et al. [22] are suggested as an effective method for quantitative analysis of exchange coupling interactions based on the equation δM = M_d_(H) − [1 − 2M_r_(H)]. The positive and negative values of δM are mainly caused by the exchange coupling effect and magnetostatic effect, respectively. Figure 5 shows the Henkel curves for the samples from different regions of the magnet. All samples show a positive peak at the magnetic field around the coercivity, indicating the existence of strong intergranular exchange coupling in all regions of the magnet. However, the value of δM varies in different regions, indicating different exchange coupling behaviors. The reasons are related to both grain size and grain boundary thickness. Based on the microstructure analysis of the magnet, the bottom and top regions have irregular grain shapes and quite thick grain boundaries. The thick grain boundary decouples the grains and reduces the intergranular exchange coupling effect, resulting in lower δM peaks. In the middle region, the grain boundaries are relatively thin, and exchange coupling is stronger, leading to higher positive δM peaks. Since coercivity can be weakened by the inter−grain exchange coupling, the middle region of the magnet exhibits relatively low coercivity, as shown in Figure 4.

### 3.4. Temperature Stability

**Figure 6:**
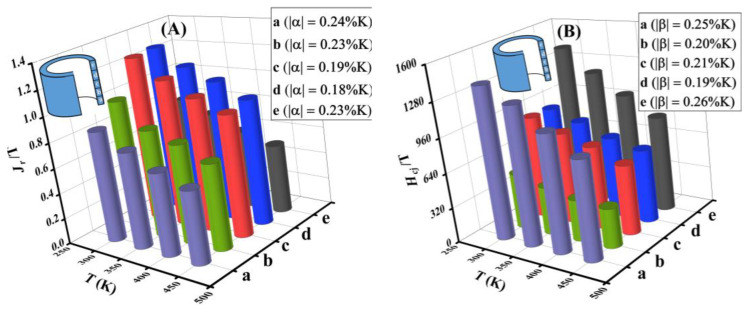
The variations of (**A**) remanence and (**B**) coercivity of the extruded ring magnet from the bottom to the top in different regions with temperature.

The temperature dependence of the remanence and coercivity in different regions of the extruded magnet are shown in Figure 6A and B, respectively. Samples a, b, c, d, and e are from the bottom to the top of the magnet. With the increasing temperature, H_cj_ and J_r_ of each region of the ring decrease, which is related to the decreasing anisotropic field and magnetization of the Nd_2_Fe_14_B phase. In addition, *H_cj_* and *J_r_* in the bottom and top regions of the ring are more temperature sensitive than those in the middle region of the ring. The temperature coefficient of remanence (α) and the temperature coefficient of coercivity (β) between 300 K and 450 K can be calculated based on the following equations:

(1) $$\alpha =\frac{J_{r}\left(T\right)-J_{r}\left(T_{0}\right)}{J_{r}\left(T_{0}\right)*\left(T-T_{0}\right)}\;\times \;100\%$$

(2) $$\beta =\frac{H_{c}\left(T\right)-H_{c}\left(T_{0}\right)}{H_{c}\left(T_{0}\right)*\left(T-T_{0}\right)}\;\times \;100\%$$

where *J_r_(T)* and *H_c_(T)* are the measured remanence and intrinsic coercivity at different temperatures, respectively. The temperature coefficients |α| and |β| for the samples from regions a, b, c, d, and e calculated by the above equations are shown in figures. Generally, the lower the absolute value of the temperature coefficient, the better the thermal stability. From the top and bottom to the middle of the magnet, the |α| value decreases from 0.24%/K to 0.18%/K, and the |β| value decreases from 0.26%/K to 0.19%/K. It indicates that the thermal stability in the middle region of the ring is higher than that at the top and bottom of the ring. Except for the initial values of room temperature coercivity and remanence, the varied thermal stability is also related to the Curie temperature. Based on the microstructure of the magnet in Figure 3, in the top and bottom regions, the Co element is segregated in the grain boundary phase. In the middle region, the Co element is partially returned to the main phase. Normally, the substitution of Fe with Co can greatly enhance the Curie temperature of the main phase, thus improving the thermal stability of the magnets [23]. Therefore, the middle region of the magnet is more thermally stable than the top and bottom regions.

### 3.5. Corrosion Resistance Properties

**Figure 7:**
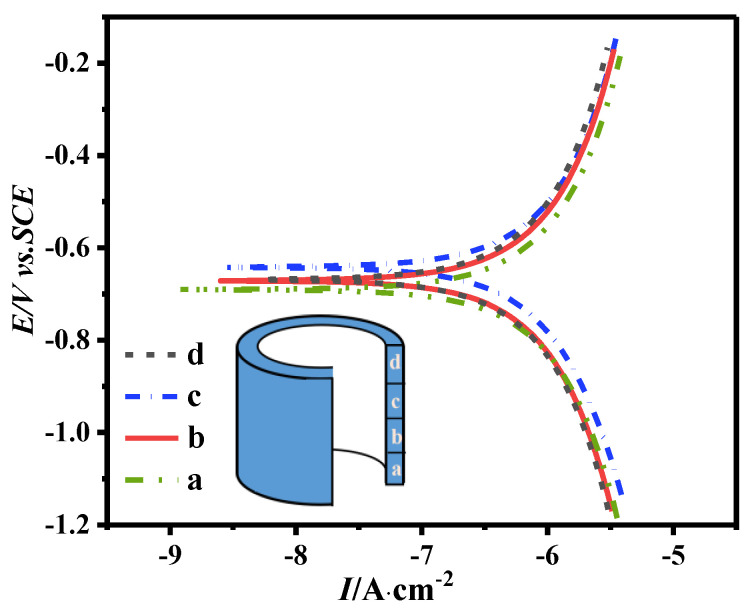
Electrochemical characteristics–polarization curves of the extruded ring magnet, where line a, b, c, and d were obtained along the radial direction from the bottom to the top in different regions.

Figure 7 shows the polarization curves obtained by electrochemical measurement in 3.5 wt.% NaCl solution for the samples from different regions of the magnet. From the top and bottom of the magnet to the middle, the polarization curves are shifted toward the upper right corner of the figure, indicating increasing corrosion potential (E_corr_) and corrosion current (I_corr_). Generally, the corrosion trend of the magnet is determined by E_corr_, and I_corr_ determines the corrosion rate [24]. The higher E_corr_ and lower I_corr_ indicate better corrosion resistance [25]. As shown in Figure 7, the middle of the ring has higher E_corr_ and equivalent I_corr_. Therefore, it is more resistant to corrosion. The reason is related to the variation of the microstructure and density of the magnet. Due to the high chemical activity of the rare earth−rich phase in NdFeB magnets, the corrosion mechanism of the magnets is intergranular corrosion [26]. In the middle region of the ring, the main grains are well–oriented and high aspect ratio platelets, with fewer but continuous and uniformly distributed grain boundary phases. At the same time, the magnet density is higher, which is beneficial to the corrosion resistance of the magnet. In the bottom and top regions, the grains are equiaxed and disordered, and the grain boundary phase is thicker and unevenly distributed with more triangular grain boundaries and more cracks, which are not beneficial to corrosion resistance. Therefore, the middle of the ring has better corrosion resistance.

### 3.6. Mechanical Properties

**Figure 8:**
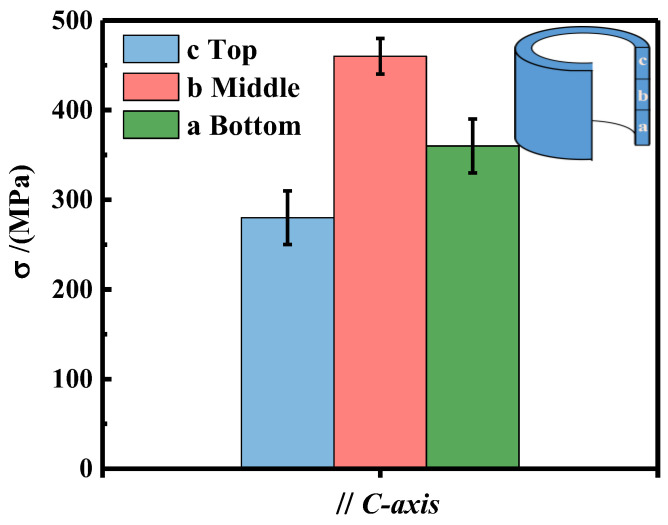
Compressive strength of the extruded ring magnet from the bottom to the top in different regions.

The mechanical properties are also important for the application of ring magnets. Figure 8 shows the compressive strength for the different regions of the magnet, and the applied force is along the direction of the *c*–axis. The result shows that the compressive strengths of the ring magnet’s top, middle, and bottom regions are 295 MPa, 465 MPa, and 355 MPa, respectively. The highest value of compressive strength is found in the middle region of the ring, which is due to the higher density and aligned grains. It is well known that the fracture behavior of hot deformed Nd–Fe–B magnets is an intergranular fracture because the strength of the Nd–rich grain boundary phase is lower than the Nd_2_Fe_14_B matrix phase [27]. For the bottom and top regions of the ring, the equiaxial grains are small and disordered. The grain boundary phase is relatively thick and unevenly distributed, resulting in the weakness of the combination strength between grains; thus, the compressive strength is low. In addition, there are more cracks and defects on the top of the ring magnet, so its compressive strength is the lowest. On the contrary, the middle region with high density and close parked platelet grains with thin grain boundaries exhibit high strength. In addition, Jin et al. [28] found that the compressive strengths of conventional hot–pressing and hot–deformed magnets along the *c*-axis are typically 700 MPa–1100 MPa. The result indicates that the mechanical properties of backward extruded NdFeB ring magnets can be further improved, possibly by optimizing the homogeneity of the microstructure.

## 4. Conclusions

In summary, we have investigated the microstructure, magnetic properties, corrosion resistance, and mechanical properties at different regions of radially oriented NdFeB ring magnets prepared from amorphous magnetic powders. The results indicate that the microstructure and properties of the magnets vary significantly in different regions along the radial direction. The grains in the middle region are well–oriented, platelet–like, and neatly arranged, with thin and continuous grain boundary phases. In the bottom and top regions, the grains are disordered and unoriented equiaxed grains with fine grains and uneven distribution of grain boundary phases. The magnetic properties with *J_r_* of 1.28 T, *H_cj_* of 920 kA/m, and *(BH)_max_* of 270 kJ/m^3^ are obtained in the middle region of the ring prepared at 750 °C with an optimized backward extrusion process. The coercivity mechanism differs in different regions of the ring magnet. The top and bottom regions are dominated by the pinning mechanism, while the middle region is jointly controlled by the pinning and nucleation mechanism. The difference in inter–grain exchange coupling leads to different magnetic properties. Various Co element segregations in the grain boundary phase and the main phase are also observed in different regions of the magnet, which influences the thermal stability. In addition, the corrosion resistance and mechanical properties of the middle region of the magnet are better than those of the top and bottom regions, which are also closely related to the microstructure. The present results show that Nd-Fe-B amorphous magnetic powder can be directly used to prepare radially oriented ring magnets, but the homogeneity of the magnet should be improved.

## Figures and Tables

**Table 1:** The average grain sizes of the extruded ring magnet in the radial direction from the bottom to the top in different regions. D_//_ and D_⊥_ are the grain sizes measured parallel and perpendicular to the *c*–plane, respectively.

Region	Average Grain Size (nm)	
	D_//_	D_⊥_
a (bottom)	123 ± 34	74 ± 20
b (middle)	336 ± 88	120 ± 32
c (top)	94 ± 30	78 ± 25

**Table 2:** Density and magnetic properties of the extruded ring magnet in the radial direction from the bottom to the top in different regions (density *ρ*, coercivity *H_c_*, remanence *J_r_*, maximum energy product *(BH)_max_*).

Properties	Region of the Ring Magnet			
	a (Bottom)	b (Middle)	c (Middle)	d (Top)
*ρ* (g/cm^3^)	7.3	7.5	7.2	6.9
*H_c_* (kA/m)	1386	930	920	1410
*J_r_* (T)	0.86	1.27	1.28	0.85
*(BH)_max_* (kJ/m^3^)	111	270	270	127

## Data Availability

Seek out the author to get the raw data.
